# Growth and Differentiation Factor 3 Induces Expression of Genes Related to Differentiation in a Model of Cancer Stem Cells and Protects Them from Retinoic Acid-Induced Apoptosis

**DOI:** 10.1371/journal.pone.0070612

**Published:** 2013-08-12

**Authors:** Karolina Tykwinska, Roland Lauster, Petra Knaus, Mark Rosowski

**Affiliations:** 1 Institute of Medical Biotechnology, Department of Biotechnology, Technische Universität Berlin, Berlin, Germany; 2 Berlin-Brandenburg School for Regenerative Therapies, Charité Campus Virchow Klinikum, Berlin, Germany; 3 Freie Universität Berlin, Institut für Chemie und Biochemie, Berlin, Germany; Virginia Commonwealth University, United States of America

## Abstract

Misexpression of growth factors, particularly those related to stem cell-like phenotype, is often observed in several cancer types. It has been found to influence parameters of disease progression like cell proliferation, differentiation, maintenance of undifferentiated phenotype and modulation of the immune system. GDF3 is a TGFB family member associated with pluripotency and differentiation during embryonic development that has been previously reported to be re-expressed in a number of cancer types. However, its role in tumor development and progression has not been clarified yet. In this study we decipher the role of GDF3 in an *in vitro* model of cancer stem cells, NCCIT cells. By classical approach to study protein function combined with high-throughput technique for transcriptome analysis and differentiation assays we evaluated GDF3 as a potential therapeutic target. We observed that GDF3 robustly induces a panel of genes related to differentiation, including several potent tumor suppressors, without impacting the proliferative capacity. Moreover, we report for the first time the protective effect of GDF3 against retinoic acid-induced apoptosis in cells with stem cell-like properties. Our study implies that blocking of GDF3 combined with retinoic acid-treatment of solid cancers is a compelling direction for further investigations, which can lead to re-design of cancer differentiation therapies.

## Introduction

Cancer stem-like cells (CSC) constitute a small population of tumor-initiating cells, with extensive self-renewal ability, capacity to generate non-tumorigenic end cells and multidifferentiation potential. CSCs are believed to be the chief cause of chemotherapy resistance and disease relapse. According to the CSC hypothesis, control over this highly proliferative cell compartment defines the ultimate cure for cancer [1]–[3].

There have been ongoing studies to define CSC markers *in vivo*, but no reliable, universal combination has been found so far. However, Ben-Porath [4] and others [5], [6] showed that a common feature of histologically poorly differentiated tumors - which generally exhibit the worst prognoses – is the ES-like signature, including expression of NANOG, OCT4, SOX2 and their targets [7], [8]. Aberrant expression of stem cell factors within tumors sustains aggressive phenotype and enhances the likelihood of progression and metastasis [9].

Among the vast amount of cancer cell lines available to study the cancer biology *in vitro*, embryonal carcinoma cell lines display ES-like signature, including the expression of main pluripotency-network associated transcription factors, and are not only able to differentiate *in vitro*, but are also highly tumorigenic *in vivo*. Therefore, embryonal carcinoma (EC) cell lines, are expected to be a suitable model of CSC [10], [11].

Growth- and differentiation factor 3 (GDF3) is widely accepted as pluripotency marker, as it is a direct transcriptional target of NANOG [12]. It was reported to regulate both major characteristics of embryonic stem cells, maintenance of the undifferentiated phenotype and of the differentiation potential [13].

Along with other embryonic stem cell markers, GDF3 was shown to be expressed in several cancer types such as breast carcinoma [14], [15], melanoma [16], seminoma [15] and testicular germ cell tumors [17]. While NANOG, OCT4 and SOX2 were identified as stemness-promoting transcription factors, the role of GDF3 remains poorly understood. Results published by others to date, regarding the putative role of this molecule in cancer biology are contradictory. It was demonstrated, that GDF3 inhibits the proliferation of breast carcinoma cell line MCF7 [14], but augments proliferation of B16 myeloma and can promote neuronal differentiation of PC12 cells [18].

GDF3 belongs to the potent growth factor family of Transforming Growth Factor β (TGFB). Members of TGFB family, including Bone Morphogenetic Proteins (BMPs), Growth and Differentiation Factors (GDFs) and TGFB, display distinct, sometimes opposing effects on target cells, depending on the cellular context, other ligands present, dosage and identity of the cytokine [19].

GDF3 was reported to be involved in both canonical pathways triggered by TGFB family members: (1) extracellular inhibition of BMPs [13] and (2) induction of SMAD2/3 phosphorylation due to binding to Activin A receptors type IB or IC (ACVRIB, ACVRIC) and Activin A receptors type IIA or IIB (ACVRIIA, ACVRIIB) in cooperation with obligatory co-receptor teratocarcinoma-derived growth factor 1 (TDGF1) [20], [21].

The emerging role of SMAD2/3 signaling cascade has been already demonstrated in pancreatic, breast, gastric, ovarian, skin and many other cancer types. However, both, activation and blocking of the SMAD2/3 pathway can have positive effects on the disease onset [22]–[24]. Therefore, the action of each ligand triggering this pathway has to be carefully investigated and evaluated.

Encouraged by these facts, we embraced the challenge to comprehensively investigate the effects of GDF3 signaling in a model of CSC line by transcriptome profiling and differentiation assays and to evaluate its potential as a therapeutic target. We found that GDF3 regulates the expression of genes involved in differentiation, but does not influence the proliferative capacity of undifferentiated CSC. Furthermore, we demonstrate that GDF3 protects the CSC from apoptosis induced by retinoic acid, the only clinically approved cyto-differentiating, anti-cancer agent.

## Materials and Methods

### Cell culture and differentiation

NCCIT and HEK293T cells (American Type Cell Collection) were grown in Dulbecco's Modified Eagle's Medium with 4.5 g/l glucose (PAA Laboratories, Austria) containing 10%FBS (PAA Laboratories, Austria) and Penicillin-Streptomycin (PAA Laboratories, Austria).

To generate GDF3 knockdown cell lines, shRNA oligonucleotides for targeting GDF3 were synthesized by TibMolBiol (TibMolBiol, Germany) and cloned into lentiviral vector pLL3.7 (addgene, USA). All constructs were sequenced (GATC, Germany) prior to application. In combination with 2 packaging plasmids PAX2 and VSVG the virus was produced by calcium phosphate transfection of 293T cells. The medium containing virus was collected, 20× concentrated in Spin® FX® UF Concentrator (Corning, Germany) and mixed with NCCIT cells with addition of 10 µg/mL polybrene (Sigma, Germany). The medium was changed to regular medium the next day.

To induce differentiation, NCCIT cells were treated with 10 µM all-trans retinoic acid (Sigma, Germany) for 14 days.

### Microarray analysis

For the transcriptome analysis human genome CGH Microarray 44K (Agilent Technologies, USA) was utilized according to manufactures protocol. In brief, the isolated RNA was labeled by Low RNA Input Fluorescent Linear Amplification kit (Agilent Technologies, USA), fragmented, mixed with control targets and hybridized overnight. The slides were then washed and scanned with 5 µm resolution using DNA microarray scanner (Agilent Technologies, USA). Features were extracted with the image analysis tool A 6.1.1. (Agilent Technologies, USA) using default settings. Data analysis was performed with Rosetta Informatics Platform Resolver Built 4.0.

The overrepresentation analysis was undertaken by uploading the gene sets with p-value≤0.05 and fold change≥1.5 to **D**atabase for **A**nnotation, **V**isualization and **I**ntegrated **D**iscovery (DAVID) v6.7 [25] and performing the Gene Onthology analysis by using the entry Panther_BP_ALL.

Microarray data (accession number GSE44670) has been submitted to the NCBI GEO database. The dataset comprises 3 conditions (A–C, described further in the GEO database and in the results part) with one biological replicate each. The untreated control in A and B is represented by a different sample.

The heat map was generated by CIMminer, a freeware developed by Genomics and Bioinformatics Group, Laboratory of Molecular Pharmacology, Center for Cancer Research, National Cancer Institute. Only probes with p≤0.05 and fold change ≥1.5 on either microarray A or B were selected. If p>0.05, fold change was default set to 1 (set to black color on the heat map).

### Isolation of nucleic acids, cDNA synthesis and qPCR

Isolation of RNA was performed using NucleoSpin® RNA II kit (Macherey-Nagel, Germany), following the instructions provided.

Reverse Transcription of mRNA was carried out by using TaqMan® Reverse Transcription Reagents cDNA kit (Applied Biosystems, USA), as per manufacturer's instructions.

Real time PCR was performed using 1 µl cDNA with 1 µl primer mix and SensiFAST™ Sybr No-ROX kit (Bioline, Germany), in 96-well PCR plates (Biozym Scientific, Germany), and were read with Stratagene MX 3005P™ Multiplex Quantitative PCR System (Agilent Technologies, USA). Primers were ordered from TIBMolBiol (Germany).

The primer list can be found in Table 1.

**Table 1:** List of primer sequences.

Primer name	upstream	downstream
GDF3	GACTGACCGCAACACAAACATT	TTCGCTTTCTCCCAGACCAA
TDGF1	GCTAACGCCTCTTTTCCCCCTA	CCCGAGATGGACGAGCAAAT
HoxA9	GAGAGCGGCGGAGACAAG	CGGTGAGGTTGAGCAGTCG
HoxA10_2	CGCAGAACATCAAAGAAGAGAGC	CTGAGAAAGGCGGAAGTAGCC
HoxB13	GTCTTGGGCTCTCGCTGGT	CGCCTCTTGTCCTTGGTGAT
TBX3	AGTCCTCCAGTGAACAAGCAG	TCAGCAGCGAAAAGGTGAG
Lefty2	GCACACCCTGGACCTCAG	TGCCCACACACTCGTAAGC
Nanog	GCGGACTGTGTGTTCTCTCAGGC	TTCCAGATCCGTTCACCAGATAG
Oct4	GACAACAATGAGAACCTTCAGGAGA	CTGGCGCCGGTTACAGAACCA
BMPR2	CATAATAGGCGTGTGCCAAAAA	GCTTGTGCTTGCTGTCGTTC
ACVRIIA	GGGAACTGGCTTCTCGCTGT	TAACCTGGCTTCTGCGTCGT
ACVRIIB	ATGCTGCCCTTTGAGGAAGA	AGTCCGAGGTAGTGCCGTTG
ACVRIB	AGGGTCGGTTTGGGGAAGTA	AGCTGTGTCCAGGTGCCATT
ACVRIC	TATGATGTGACCGCCTCTGG	TCTGCCTCACGAAACCAAGA
NODAL	GAGGAGTTTCATCCGACCAACC	GAGGCACCCACATTCTTCCAC
SMAD2	GCCGCCAGTTGTGAAGAGAC	TGGAGACGACCATCAAGAGACC
SMAD3	GCTGACACGGAGACACATCG	AGCCTCAAAGCCCTGGTTG
SMAD4	CTTTGAGGGACAGCCATCGT	GCCACAGGAATGTTGGGAAA
SMAD7	GCTGAAACAGGGGGAACGA	AGTATGCCACCACGCACCA
Grem2	TTCCCTGTCCTTGTTCCTGGT	CCTCCTCGCTCACCGTCT
GAPDH	TGTTGCCATCAATGACCCCTT	CTCCACGACGTACTCAGCG
Pax6	CCTACCACAGCCCCAAGGT	AGCAACATAACCAGAAGGAGCAG
Nestin	GGCAGCGTTGGAACAGAGGTTGGA	CTCTAAACTGGAGTGGTCAGGGCT
LC Bmp2	CCTCATCCCAGCCCTCTGAC	GGTTGTTTTCCCACTCGTTTCTG
BMP4 LC	CGGGATCTTTACCGGCTTC	TCTGCTGGGGGCTTCATAAC
LC BMP7	ACTGTGAGGGGGAGTGTGC	CGAAGTAGAGGACGGAGATGG
Tie2	TGTTCCTGTGCCACAGGCTG	CACTGTCCCATCCGGCTTCA
TUBB3	CAGCAAGGTGCGTGAGGAG	TGCGGAAGCAGATGTCGTAG
TDGF1 cloning	ATTAGCTAGCTGGAAACTGATCTTCAATGCAC	ATTAGGATCCTTCACCCAGTGCTTCAGCTT

### Luciferase assay

The BMP-responsive construct BRE-luc [26] and SMAD binding element construct SBE-luc were kind gifts from Prof. P. Ten Dijke (Leiden University Medical Center). CAGA-luc was a kind gift from Prof. P. Knaus (Freie Universität Berlin). The cells were seeded, transfected with TurboFect (Thermo Scientific, Germany, procedure according to the manufacturers protocol) and 24 h later starved for 3 h in serum-free medium and subsequently stimulated for at least 20 h with ligand of interest (rhGDF3, rhNodal and rhBMP2 were purchased from R&D Systems, Germany). To test the inhibitory capacity of GDF3, rhGDF3 and rhBMP2 were incubated together in serum-free medium for 1 h before cell stimulation. The cells were then lysed and the luciferase activity was measured in lysates by Dual Luciferase® Reporter Assay System (Promega, Germany) in microplate reader Omega (BMC Labtech, Germany). Data is represented as relative luciferase units (RLU), which are calculated as firefly to renilla ratio.

### TDGF1 expression plasmid

TDGF1 expression plasmid was created by amplification of complete TDGF1 ORF using NCCIT cDNA as a template with primers TDGF1 cloning up/TDGF1 cloning do. The PCR product was inserted into pIRES2-EGFP (BD Biosciences, Germany) via NheI/BamHI restriction sites. NheI, BamHI, Taq Polymerase and T4 Ligase were purchased from Thermo Scientific.

Primer sequences are listed in the Table 1.

### Immunofluorescence

The cells were seeded in chamber slides (BD Biosciences, Germany) and on the following day fixed in 4%PFA, permeabilized with PBS/0.05%Triton X-100 (Sigma, Germany) and stained according to the protocol of ab supplier. The applied antibodies were: mouse anti-human SMAD2 ab (Thermo Scientific, Germany), rabbit anti-human TUBB3 ab (Sigma, Germany) and anti-mouse IgG (H+L) –A594 ab and anti-rabbit IgG (H+L) - Alexa594 ab (Molecular Probes, USA).

For the SMAD translocation, cells were starved 3 h in serum-free medium and subsequently stimulated with 300 ng/mL rhGDF3 for 1 h, prior to fixation.

The photographs were acquired with a digital fluorescence microscope BZ 9000 (Keyence, Germany) and visualized with BZviewer (Keyence, Germany), after counterstaining the nuclei with Hoechst 33342 (Sigma, US).

### Western Blot

For western blot analysis, cells were lysed in 5xLaemmli buffer and boiled for 5 min. Whole cell lysates were separated by 10% SDS–polyacrylamide gel electrophoresis and proteins were transferred to a PVDF membrane (Macherey-Nagel, Germany). Membranes were blocked in 5% BSA and then incubated with an anti-SMAD2 (Thermo Scientific, Germany), anti-pSMAD2 (Invitrogen, Germany), and anti-GAPDH (Thermo Scientific, Germany) abs, followed by incubation with a horseradish peroxidase-conjugated secondary abs (Thermo Scientific, Germany). Immunoreactive proteins were visualized using an enhanced chemiluminescence detection kit (Thermo Scientific, Germany). The photographs of the membranes were acquired by Fusion Fx7 (Peqlab, Germany).

### FACS Analysis

Proliferation was tracked by staining of the cells with CellTrace® Violet (Invitrogen, Germany) and apoptosis by Annexin V- Pacific Blue (BioLegend, USA) according to the manufacturer's protocol.

Flow cytometry analysis was carried out using a MACSQuant® Analyzer (Miltenyi, Germany) flow cytometer and the data was analyzed using FlowJo software, version 7.6.5 (Tree Star inc., USA). Data from at least 30 000 cells was routinely acquired for each sample. Cell count has been normalized to the peak height at mode of the distribution by FlowJo algorithm, so that absolute count is represented by 100% of total (% of Max).

### Statistical analysis

Unpaired student t-test was applied to the data sets, using *GraphPad Prism*® software version 5.0 (GraphPad Software Inc., USA). P-values smaller than or equal to 0.05 were considered significant. (*) indicates p≤0.05, (**) p≤0.01 and (***) p≤0.001. Data is represented as means +/− standard deviation.

## Results

### NCCIT cells express essential components of the GDF3 signaling cascade

**Figure 1:**
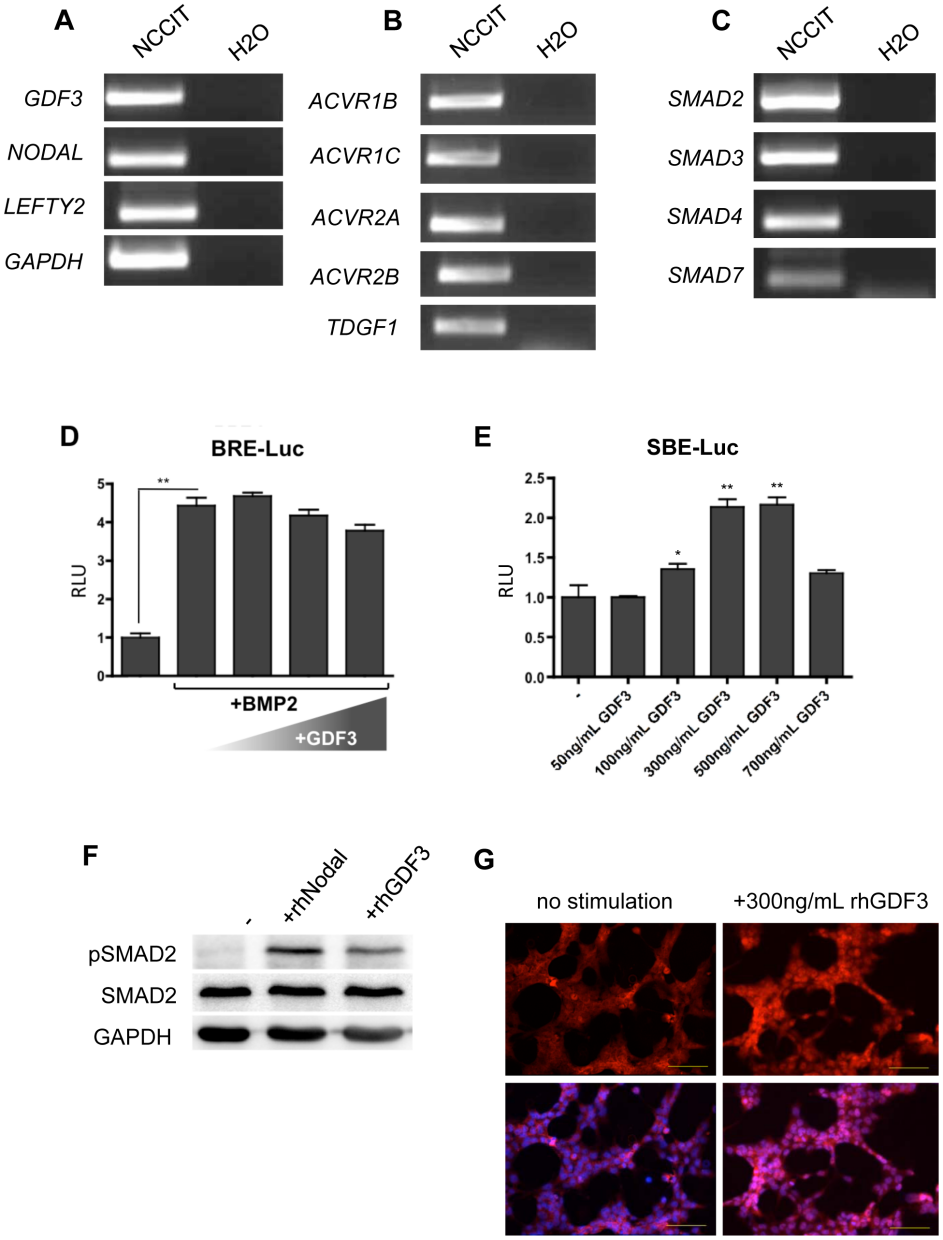
GDF3 signals via SMAD2/3 pathway in NCCIT cells. **A–C.** Expression of GDF3, agonistic ligand NODAL and extracellular inhibitor Lefty2 (A), GDF3 receptors (B) and intracellular signaling mediators SMADs (C). The expression was determined by RT-PCR, GAPDH expression served as a control. One representative example is depicted. n = 3 **D.** BRE-dependent luciferase activity in NCCIT cells treated with 20 ng/mL BMP2 alone as a positive control or with pre-incubated mixtures of 20 ng/mL BMP and 3×, 5×, 10× molar excess of GDF3. **E.** SBE-dependent luciferase activity in NCCIT cells stimulated with GDF3 in concentrations ranging from 50 to 700 ng/mL. The results in D–E are showed as a firefly to renilla ratio and normalized to non-stimulated sample. The bars represent a mean value of three biological replicates +/− standard deviation. P-values smaller than or equal to 0.05 were considered significant. (*) indicates p≤0.05, (**) p≤0.01. **F.** Immunoblot analysis of SMAD2 phosphorylation in NCCIT cells after starvation and treatment with 300 ng/mL NODAL or 100 ng/mL GDF3 for 1 h. GAPDH was used as a loading control. One representative immunoblot is depicted. n = 3. **G.** Translocation of SMAD2 in NCCIT upon stimulation with 300 ng/mL GDF3. The cells were stained with anti-SMAD2 and anti-mouse-Alexa594 (upper panel) antibody and counterstained with Hoechst (lower panel). Yellow bar indicates 100 µm. One representative example is depicted. n = 3.

GDF3 and its obligatory co-receptor TDGF1 have a narrow expression pattern and are associated with pluripotent phenotype in embryonic stem cells and cancer. To establish the embryonal carcinoma cell line NCCIT as a suitable CSC model to study the role of GDF3, we evaluated the expression of important components of GDF3 signaling pathway by RT-PCR. We detected expression of *GDF3* and other secreted ligands potentially involved in GDF3 signaling, such as *NODAL* and *LEFTY2* (Figure 1A). NODAL utilizes the same type I and type II receptors [27] and can therefore compete with GDF3 for the receptor binding sites. LEFTY2 is a natural, extracellular inhibitor of NODAL and GDF3 [28]. Also transcripts of both, type I (ACVRIB and C) and type II (ACVRIIA and B) receptors are present in NCCIT cells, along with the obligatory co-receptor *TDGF1* (Figure 1B) and the intracellular signaling mediators R-SMADs (*SMAD2* and *SMAD3*), co-SMAD (*SMAD4*) and the inhibitory *SMAD7* (Figure 1C), involved in the negative feedback loop of SMAD2/3 signaling cascade in embryonic stem cells.

The expression of GDF3 ligand, receptors and effectors was tested in another embryonal carcinoma cell line, NTERA2 (Figure S1). Except of *ACVRIC* and *ACVRIIB*, all components of GDF3 signaling cascade were also detected by RT-PCR.

### GDF3 signaling is functionally active in the CSC model

GDF3 was reported to (1) be able to block SMAD1/5/8-signaling by binding to BMPs, e.g. BMP4 in the extracellular space [13] and (2) induce SMAD2/3 signaling cascade [20], [29]. In the latter case GDF3 acts by binding to and joining cell surface receptors TDGF1 and dimers of type I and type II receptors that leads to phosphorylation of SMAD2 or SMAD3 as intracellular effectors that are subsequently translocated into the cell nucleus to act as co-transcription factors. To test which mode of action of GDF3 is functional in our CSC model, we performed a luciferase assay. The NCCIT cells were transfected with vectors containing BMP-responsive elements (BRE-Luc) and subsequently stimulated with a mixture of BMP2 and GDF3 in 1-, 3-, or 10-fold molar excess. As shown in Figure 1D, the luciferase expression was robustly activated by BMP2, despite GDF3 presence in the medium.

To assess the activation of SMAD2/3 pathway, we transfected the NCCIT cells with a vector containing the SMAD Binding Element (SBE-Luc) and stimulated the cells with increasing concentrations of GDF3. The maximal luciferase activity driven by the promoter construct was achieved with 300 ng/mL and did not increase any further with increasing concentrations of the ligand (Figure 1E).

We confirmed the identity of phosphorylated SMAD by protein extraction from GDF3-stimulated NCCIT cells and detection of phosphorylated SMAD2 by western blot. Stimulation with recombinant human NODAL (rhNodal) was performed as a positive control for activation of SMAD2/3 signaling cascade (Figure 1F). We were also able to track the translocation of SMAD2 into the nucleus upon GDF3-stimulation by immunofluorescence staining, shown in Figure 1G.

In summary, we were able to show that GDF3 induces the SMAD2/3 pathway in NCCIT cells, and does not function as an extracellular BMP-antagonist.

### GDF3 does not influence proliferation of NCCIT cells

**Figure 2:**
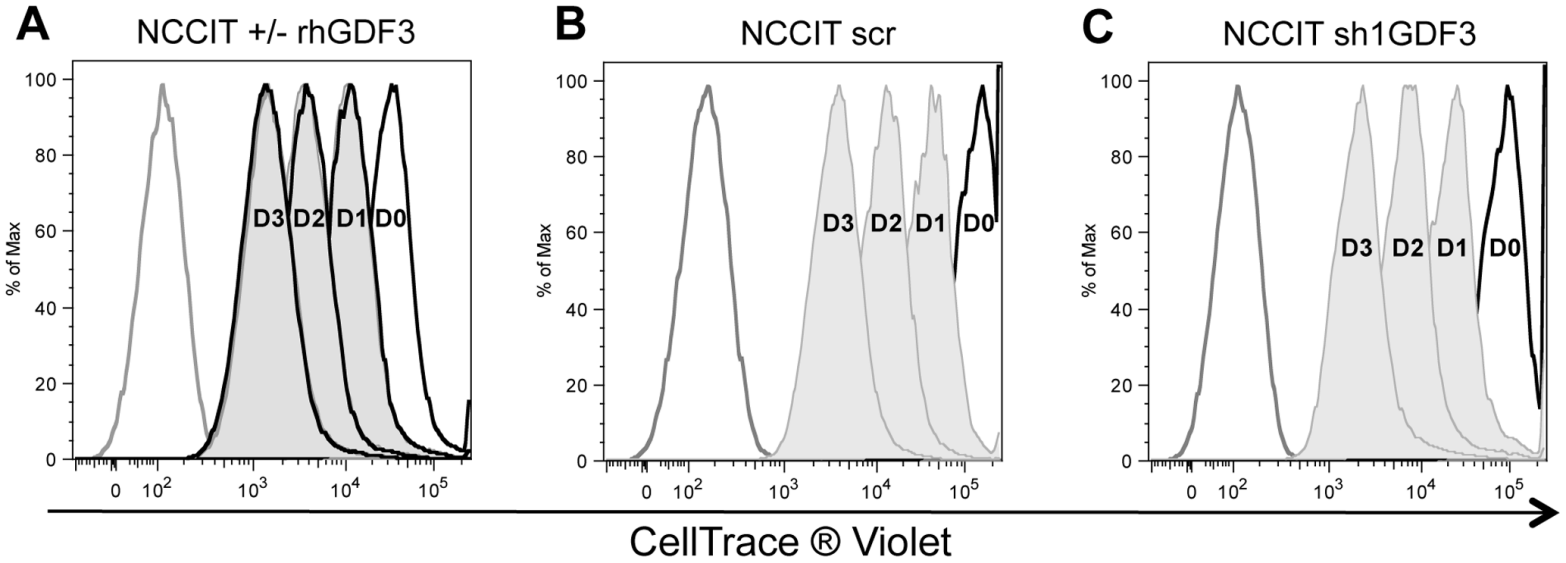
GDF3 does not influence proliferation of NCCIT cells. **A.** FACS analysis of the amount of CellTrace® Violet incorporated into NCCIT cells repetitively stimulated with 300 ng/mL rhGDF3 (tinted curve) and untreated (black line). **B–C.** FACS analysis of the amount of CellTrace® Violet incorporated into NCCIT scr (B) and NCCIT sh1GDF3 (C) cells. The cells were stained, seeded and harvested each day (indicated as D0–D3). Grey line indicates unstained cells. Cell count has been normalized to the peak height at mode of the distribution by FlowJo algorithm, so that absolute count is represented by 100% of total (% of Max). The experiment was performed in triplicate, one representative example is depicted.

Since GDF3 was reported to influence proliferation of cancer cells previously [14], [16], we determined whether GDF3 has an impact on proliferation of the CSC model cell line. The NCCIT cells were stained with a CellTrace® Violet dye, seeded and stimulated with rhGDF3 every 24 h. The cells were harvested every day and the amount of the incorporated dye into GDF3-stimulated and untreated cells was assessed by FACS and compared. No differences were found, as shown in Figure 2A and Table 2.

**Table 2:** List of the normalized differences of mean fluorescence intensity peaks.

normalized difference of mean fluorescence intensity	NCCIT			NCCIT+rhGDF3		
D0–D1	0.641	+/−	0.015	0.642	+/−	0.005
D1–D2	0.641	+/−	0.018	0.657	+/−	0.006
D2–D3	0.623	+/−	0.003	0.583	+/−	0.021

normalized difference of mean fluorescence intensity	NCCIT scr			NCCIT sh1GDF3		
D0–D1	0.677	+/−	0.003	0.693	+/−	0.006
D1–D2	0.671	+/−	0.001	0.667	+/−	0.008
D2–D3	0.710	+/−	0.003	0.699	+/−	0.006

The values were normalized to the mean fluorescence value of the peak representing the previous day. Upper panel represents quantification of the Figure 2A, the lower one of the Figures 2B–C. D0/D1/D2/D3 stands for mean fluorescence intensity measured on day 0/1/2/3. No significant differences between either rhGDF3-stimulated and untreated NCCIT cells, or NCCIT scr and NCCIT sh1GDF3 could be detected.

To test the effect of decreased concentration of endogenous GDF3 in NCCIT cells, we created a stable GDF3 knockdown cell line, by transduction of NCCIT cells with lentivirus with a shRNA-cassette targeting GDF3. The efficiency of the transduction and subsequent GDF3 downregulation was monitored via FACS by GFP expression and by qPCR for *GDF3* expression, respectively (Figure S2). Of the two shRNA constructs tested, GDF3 knockdown in the cell line generated with sh1GDF3 was more efficient and reached 96%, and was therefore employed for all following experiments. GDF3 knockdown did not influence the proliferative capacity of NCCIT cells, when compared to control cells, transduced with scrambled vector (Figure 2B–C and Table 2).

### GDF3 modulates gene expression in the CSC model

To get more insight into the potential role of GDF3 in our CSC model and to identify GDF3-downstream targets, global gene expression profiles of NCCIT cells stimulated with GDF3 or with GDF3 knockdown were analyzed by a cDNA microarray platform. We chose a short stimulation period of 3 h, to assess primary effects of the ligand stimulation.

The transcriptional response to stimulation by various TGFB family members that signal via the same pathway often varies, depending in the first place on the strength of SMAD-signaling triggered in target cells and activation of non-SMAD pathways [30]. Additionally, ligand concentration-dependent SMAD2/3 signaling was reported to trigger differential, even opposing effects [19], [31]. Therefore, in our study we aimed to cover a broad range of transcriptional effects caused by decreasing and increasing the strength of SMAD2/3 signaling in NCCIT cells (Table 3, microarrays A to C). Specifically, we applied low (100 ng/mL) and high (300 ng/mL) dose of GDF3 (microarray A & B, respectively). On the microarray C we investigated the effects of GDF3 knockdown in the CSC model cell line.

**Table 3:** Regulated genes on microarrays A–C with a p-value (p)≤0.05 and fold change (FC)≥1.5.

		p≤0.05	FC≥1.5	# of genes with official gene annotation	# of up-/down-regulated genes		# of genes mapped and annotated by DAVID
A	300 ng/mL GDF3 vs. untreated	1287	778	421	↑up	247	385
					↓do	174	
B	100 ng/mL GDF3 vs. untreated	1348	891	390	↑up	201	370
					↓do	189	
C	GDF3 KD vs. scrambled	5395	3291	2075	↑up	834	1827
					↓do	1241	

KD stands for knockdown. n = 1.

For the evaluation of microarray data, only spots with a p-value ≤0.05, fold change ≥1.5 and with official gene annotation were included (Table 3). This narrowed the amount of differentially expressed genes to 390 and 421 due to stimulation with low and high dose of GDF3. The number of regulated transcripts positively correlated with the strength of SMAD2/3 signaling induced by the applied ligand(s) (compare Figure 1E). The most prominent transcriptional changes with 2075 differentially regulated genes were observed as a result of GDF3 knockdown. While due to GDF3 treatment more genes were upregulated than downregulated (microarrays A & B, Table 3), the opposite pattern is displayed as a result of GDF3 knockdown (microarray C, Table 3).

### GDF3 acts in a dose-dependent manner

To address the biological role of GDF3, genes regulated due to GDF3 stimulation and knockdown (Table 3) were categorized on the basis of their biological function. For this purpose regulated genes with official gene names were extracted from each microarray and an overrepresentation analysis was performed by **D**atabase for **A**nnotation, **V**isualization and **I**ntegrated **D**iscovery (DAVID) v6.7 [25]. This procedure allows evaluating whether a particular functionally defined group of genes is represented more than expected by chance within a gene list [32]. Genes regulated by GDF3 were classified to main categories such as developmental processes, mesoderm and ectoderm development, neurogenesis and signal transduction (Table S1 and Table 4). Between 15.3% and 18.2% of genes regulated on microarrays A and C, respectively, were associated with developmental processes. Regulation of genes with function in ectoderm development and neurogenesis was noted on microarrays A (6.5% and 6.5%) and C (6.5% and 5.8%), but not B. Furthermore, among genes regulated by GDF3 knockdown 5,3% were associated with mesoderm development. Approx. 25% of genes on all microarrays were classified to signal transduction. Overrepresetation analysis revealed also that a small, but significant number (p≤0.05) of genes related to angiogenesis on microarray C (0.9%) (Table S1) is regulated by SMAD2/3 signaling.

**Table 4:** Overrepresentation analysis of genes regulated on microarrays A–C.

	Stimulation/knockdown		Developmental processes	Mesoderm development	Ectoderm development	Neurogenesis	Signal transduction
A	300 ng/mL GDF3	# genes	59	ns.	25	25	103
		%	15.3	ns.	6.5	6.5	26.8
		p-Value	2.30E-02	ns.	1.10E-02	1.80E-03	4.70E-05
B	100 ng/mL GDF3	# genes	ns.	ns.	ns.	ns.	85
		%	ns.	ns.	ns.	ns.	23.1
		p-Value	ns.	ns.	ns.	ns.	6.40E-03
C	GDF3 KD	# genes	329	95	117	105	415
		%	18.2	5.3	6.5	5.8	23
		p-Value	1.40E-21	2.90E-08	4.10E-10	4.70E-10	4.40E-09

Genes regulated on the microarrays A–C were assigned to biological processes. A selection of biological processes is depicted. Non-significant enrichment (p-value >0.05) is indicated as “ns.”

KD stands for knockdown.

**Figure 3:**
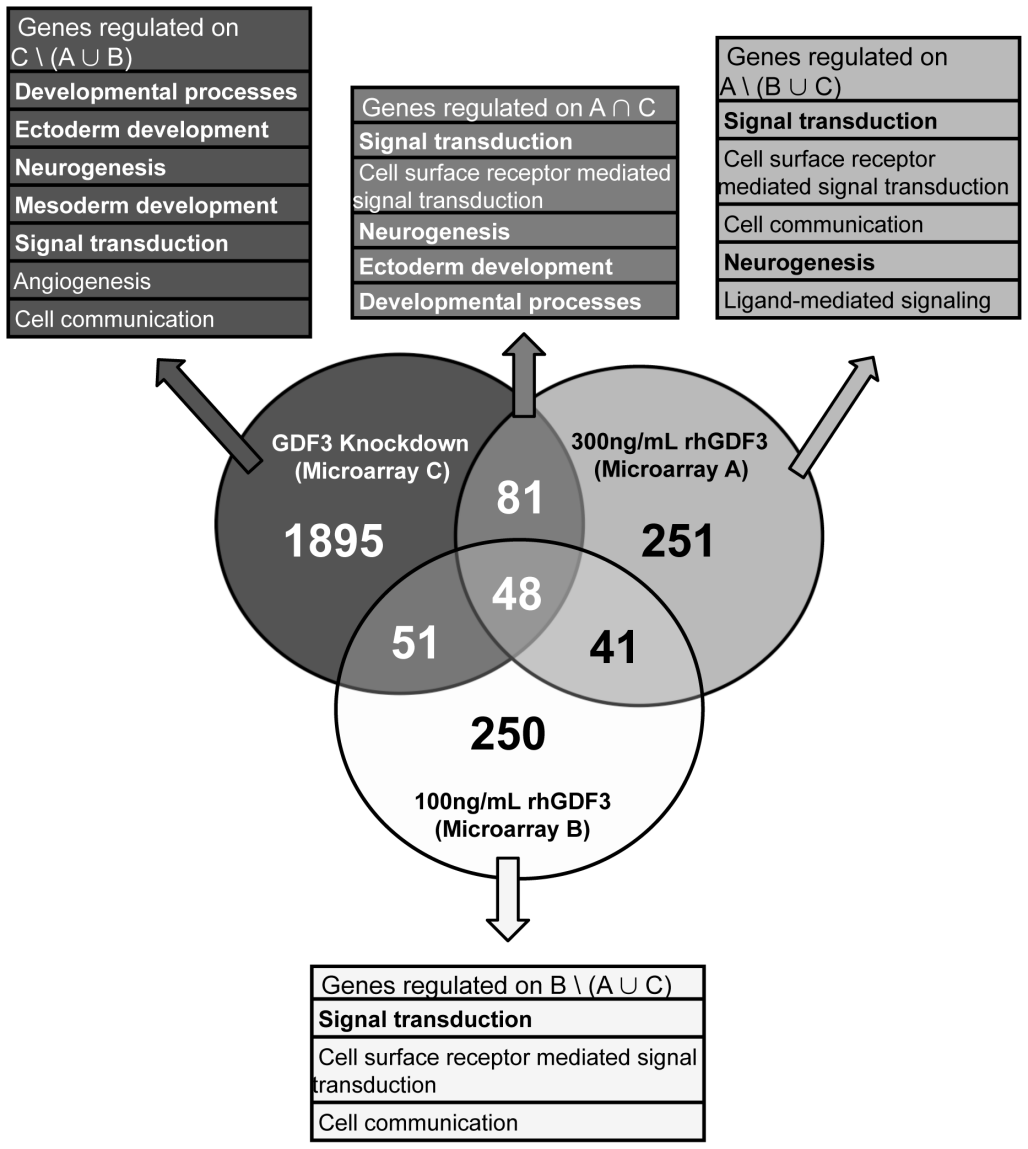
GDF3 affects expression of genes related to development and signal transduction. Venn diagram illustrating the overlap between microarrays A, B and C. Only probes on the microarrays with p-value ≤0.05 and fold change ≥1.5 were used in this analysis. Subsets of genes indicated in the headings of the color-coded boxes were subjected to the gene ontology overrepresentation analysis and a selection of predominantly enriched biological processes is listed (complete list can be found in Table S1). The biological processes listed also in the Table 4 are printed bold. A – stimulation with 300 ng/mL rhGDF3, B – stimulation with 100 ng/mL rhGDF3, C – GDF3 knockdown.

The stimulation of NCCIT cells with different concentrations of GDF3 and GDF3 knockdown differentially affected SMAD2/3 signaling strength (Figure 1E). To further compare these effects, we analyzed the quantitative overlap of transcriptional changes in stimulated cells as well as the subsets of genes exclusively regulated by each experimental condition (Figure 3). The extracted gene lists were subsequently subjected to the overrepresentation analysis. 48 genes were commonly regulated in all three conditions. However, only 9 of them were significantly enriched according to their biological function to the category of cell surface receptor mediated signal transduction. Of the 2075 and 421 genes differentially regulated by GDF3 knockdown or high concentration of GDF3, respectively (A & C), 129 transcripts were regulated in both conditions. These were predominantly enriched in categories related to development and lineage commitment: developmental processes, ectoderm development and neurogenesis. However, when commonly regulated genes were excluded from the gene pool and only genes regulated exclusively by increased (microarray A) or disrupted (microarray C) SMAD2/3 signaling were analyzed, major differences in the impact on several biological processes were observed. While the high dose of GDF3 impacted only processes related to signaling (cell surface receptor mediated signal transduction, cell communication, ligand-mediated signaling, signal transduction) and neurogenesis, the GDF3 knockdown affected further sets of genes assigned not only to signaling, developmental processes and ectoderm development, but also mesoderm development and hematopoiesis.

**Figure 4:**
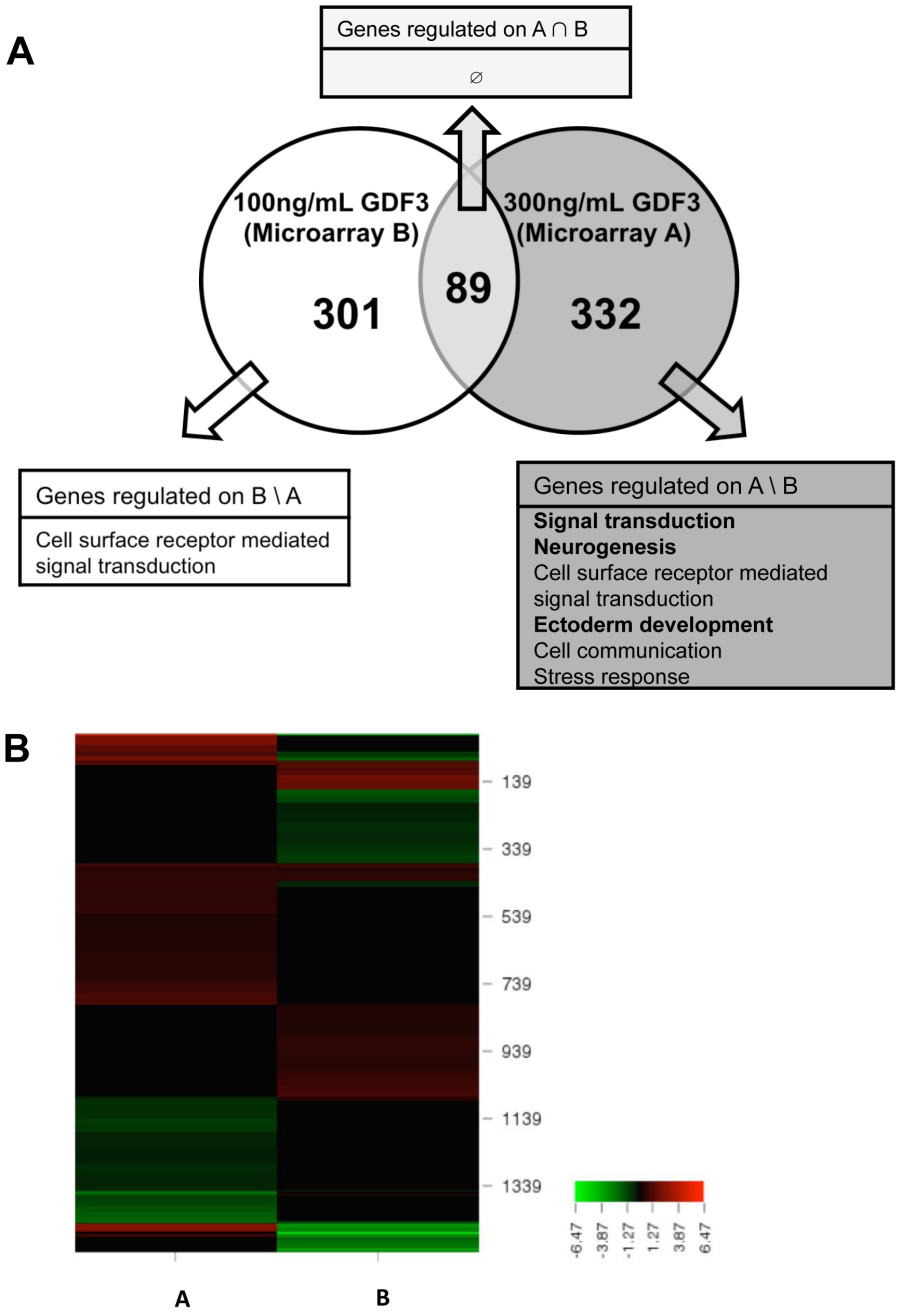
GDF3 induces distinct transcriptional response in a dose-dependent manner. A. Venn diagram illustrating cross-analysis of commonly regulated genes due to 300 ng/mL rhGDF3 (Microarray A) and 100 ng/mL rhGDF3 (Microarray B) stimulation of NCCIT cells. Only genes with p-value ≤0.05 and fold change ≥1.5 were included. B. Heat map of fold changes of genes regulated by high and low dose of GDF3 (Microarray A & B). Genes with fold change ≥1.5 and p-value ≤0.05 on at least one of the microarrays were included. Black bars indicate fold change <1.5 or p-value >0.05. The fold change is color-coded from green to red (see scale bar).

A similar procedure was employed to analyze the function of genes modulated by high and low doses of GDF3 (Figure 4A). 89 genes were regulated in both conditions, but assessment of gene ontology attributes within this group delivered no statistically significant (p≤0.05) results. We noted clear differences in biological processes regulated by different concentrations of GDF3. High GDF3 concentration impacted transcription of several genes associated with ectoderm development, neurogenesis and signal transduction, while 100 ng/mL of GDF3 modulated only process of signal transduction. The heat map of fold changes highlights several groups of genes potentially regulated differentially (Figure 4B) by high and low concentration of GDF3.

The three conditions applied to dissect the impact of GDF3 on NCCIT cells comprising (A) high dose of GDF3 to maximally activate SMAD2/3 pathway, (B) low dose of GDF3 for moderate SMAD2/3 signaling and (C) disruption of GDF3 signaling by GDF3 knockdown generated different levels of GDF3 signaling strength. Our results indicate that modulation of SMAD2/3 signaling by GDF3 stimulation or disruption of GDF3 signaling pathway affects several biological processes such as developmental processes, ectoderm development, neurogenesis and signal transduction on the transcriptional level. Additionally, the stable GDF3 knockdown led to induction of genes associated with mesoderm development and hematopoiesis in NCCIT cells. Furthermore, GDF3 modulated the transcriptome of NCCIT cells in a dose-dependent manner. Even a low dose of GDF3 (microarray B) significantly changed the transcriptome of the target cells. However, in contrary to a high dose of GDF3, a low dose did not have any impact on regulation of genes associated with development.

### GDF3 induces expression of various genes associated with signal transduction and development

To confirm microarray results, we chose several genes related to signal transduction and developmental processes and validated their expression upon GDF3 stimulation by qPCR. The compilation of microarray and qPCR results is presented in the Table 5, summarizing the validation of the microarray experiment.

**Table 5:** List of fold changes of selected genes on the microarrays A–B and qPCR.

	Microarray		qPCR	
Gene name	A	B	300 ng/mL rhGDF3	100 ng/mL rhGDF3
LEFTY2	5.8	ns.	32	5.7
GREM2	3.05	ns.	5.1	2.2
BMPR2	1.68	ns.	1.62	ns.
HOXA9	18.7	ns.	15.9	3.4
HOXA10	2.6	ns.	9.2	2.3
HOXB13	2.1	1.77	4.1	1.65
TBX3	1.86	ns.	2.3	1.5

**Figure 5:**
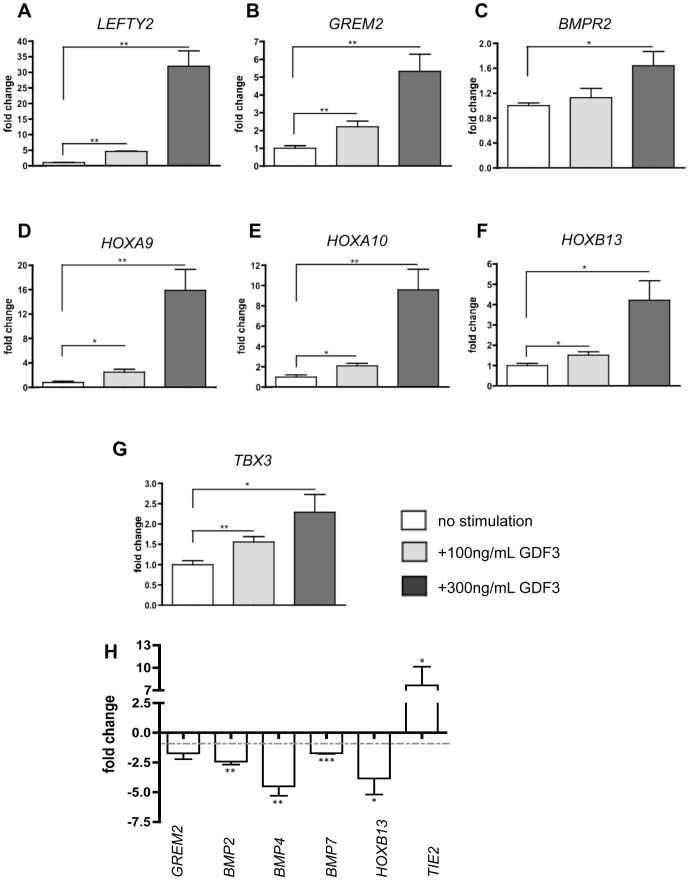
GDF3 induces expression of various genes associated with signal transduction and development. **A–G.** Effect of stimulation with different GDF3 concentrations on transcription of several genes regulated on the microarrays A & B. n = 5. **H.** Effect of GDF3 knockdown on transcription of several genes regulated on the microarray E. n = 3. The expression was measured by qPCR, the results are presented as *GAPDH* ratio and normalized to unstimulated cells (A–G) or cells transduced with scrambled-vector (H). P-values smaller than or equal to 0.05 were considered significant. (*) indicates p≤0.05 and (**) p≤0.01. Data is represented as means +/− standard deviation.

The microarray results represent n = 1, while the qPCR results represent a mean value of 5 measurements (compare Figure 5). Not significant values (p-value >0.05) are indicated as ‘ns.’

Choosing candidates for GDF3 target genes we focused mainly on members of TGFB family and transcription factors, which can act as main switches in cell fate decisions.

The expression of *LEFTY2*, a known SMAD2/3 target in embryonic stem cells [33], was strongly upregulated by low and high dose of GDF3 (Figure 5A) and served as a positive control for the stimulation.

Gremlin 2 (GREM2) and BMP receptor type 2 (BMPR2), molecules involved in BMP signaling, were induced upon GDF3 stimulation (Figure 5B–C), indicating that GDF3 has an impact on SMAD1/5/8- signaling pathway.

Stimulation with GDF3 robustly activated expression of transcripts from Hox-family, inducing 16-fold increase in *HOXA9*, 9-fold increase in *HOXA10* and 4-fold increase in *HOXB13* expression (Figure 5D–F). We could also validate induction of *TBX3* (Figure 5G), which role in neuroepithelial differentiation in hESC was recently described [34].

Validation of microarray E revealed reciprocal regulation of several genes found to be induced by GDF3 stimulation. *GREM2* was 2-fold and *HOXB13* 4.5-fold downregulated in GDF3-knockdown NCCIT cells (Figure 5H). We also found several BMP-ligands, such as *BMP2*, *4* and *7* to be downregulated due to decreased GDF3 expression. Moreover, the expression of *TIE2*, an angiopoietin 1 and 2 receptor, was 7-fold increased upon GDF3 knockdown.

Briefly, we were able to verify the microarray results and thereby define new potential GDF3 target genes.

### Blocking of ACVRIB and ACVRIC abrogates induction of novel GDF3-downstream target genes

**Figure 6:**
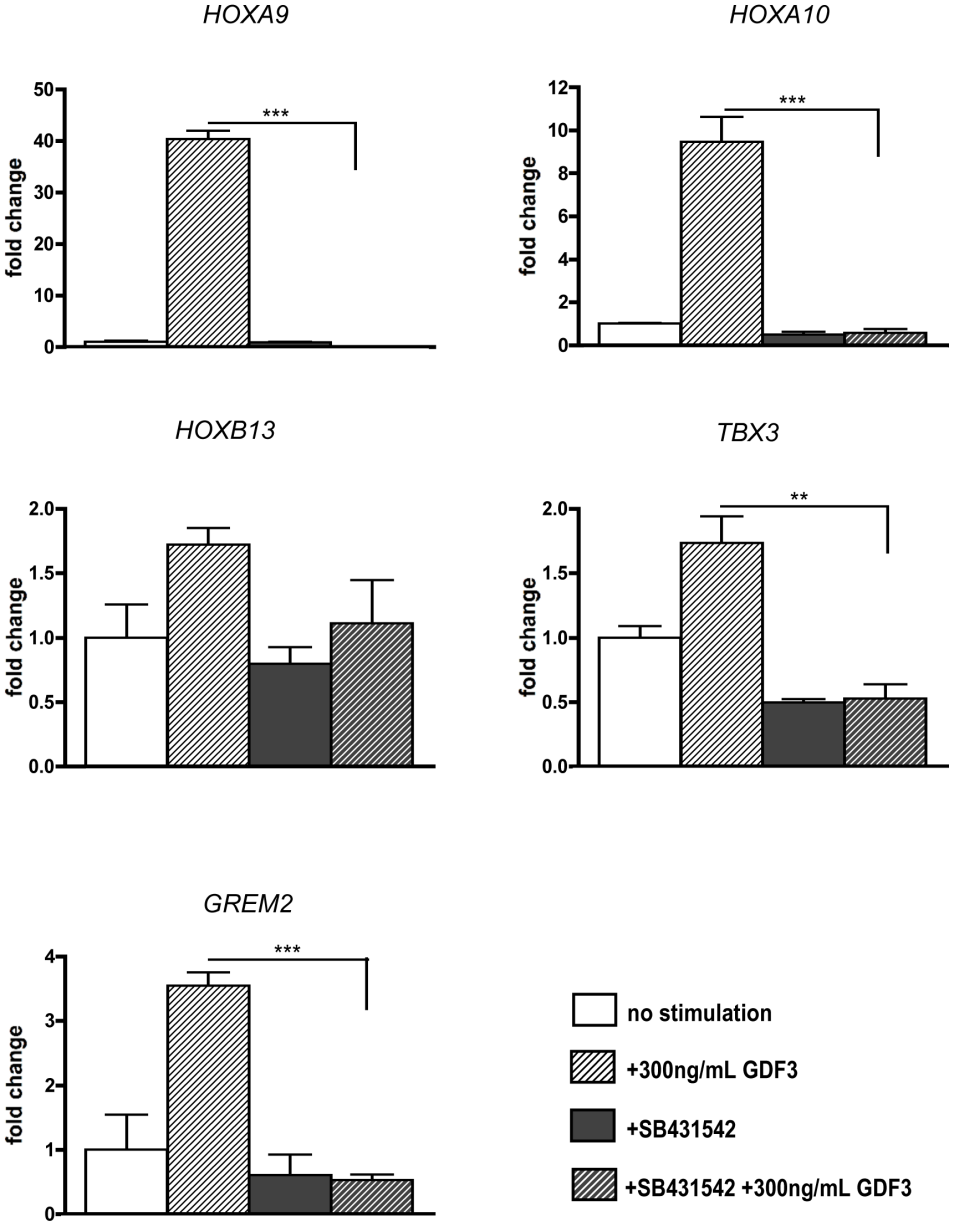
Induction of GDF3-downstream target genes is ACVRIB-dependent. Effects of GDF3 and ACVRIB-inhibitors SB431542 on the expression of HOXA9, HOXA10, HOXB13, TBX3 and GREM2. The expression was measured by qPCR, the results are presented as *GAPDH* ratio and normalized to untreated cells. P-values smaller than or equal to 0.05 were considered significant. (*) indicates p≤0.05, (**) p≤0.01 and (***) p≤0.001. Data is represented as means +/− standard deviation. n = 3.

As we observed induction of several genes upon GDF3 stimulation, it is important to verify, that the effects are GDF3 specific. For this purpose, we stimulated the cells along with addition of the ACVRIB, ACVRIC and TGFBRI–inhibitor, SB431542. The inhibitor completely abolished induction of investigated potential GDF3-target genes like *HOXA9*, *HOXA10*, *TBX3*, and *GREM2* (Figure 6). In the case of *HOXA10* and *TBX3* addition of SB431542 caused reduction of endogenous level of the respective molecule.

### GDF3 influences early differentiation events

**Figure 7:**
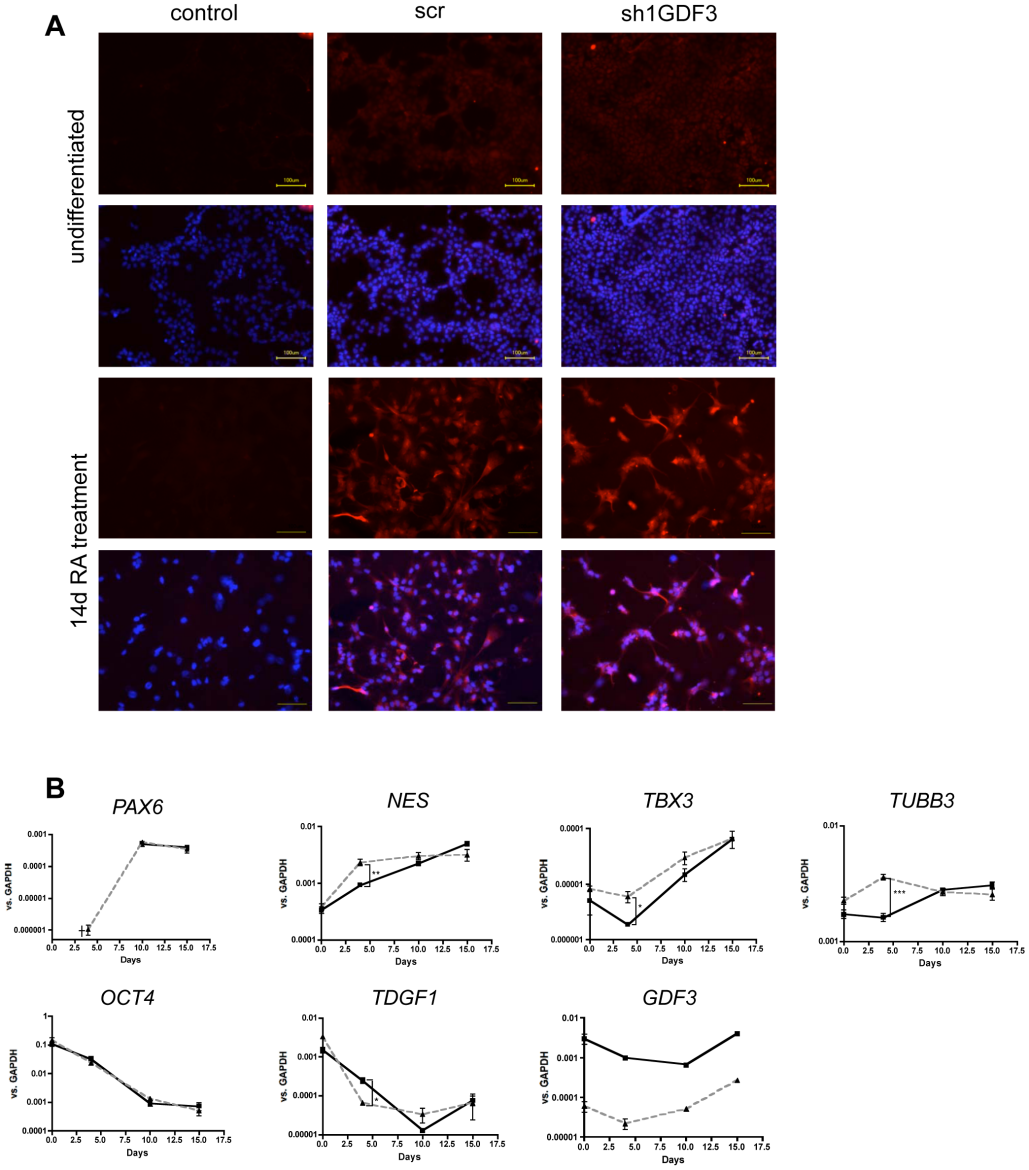
GDF3 knockdown alters the early course of neuronal differentiation. **A.** TUBB3-immunofluorescence staining of undifferentiated and 14 days RA-differentiated NCCIT scr and NCCIT sh1GDF3 cells. The cells were stained with anti-TUBB3 and anti-mouse-Alexa594 (upper panel) antibody and counterstained with Hoechst (lower panel). Yellow bar indicates 100 µm. One representative example is depicted. n = 3 **B.** Expression of neuronal markers (upper panel) and pluripotency markers (lower panel) during RA-differentiation. The expression was measured by qPCR, the results are presented as *GAPDH* ratio and normalized to untreated cells. P-values smaller than or equal to 0.05 were considered significant. (*) indicates p≤0.05 and (**) p≤0.01. Data is represented as means +/− standard deviation. n = 3.

The microarray data clearly hinted at involvement of GDF3 in the signal transduction and differentiation events. Since NCCIT cells were reported to differentiate *in vitro* into neuronal lineage, we tested the influence of GDF3 on this process. We differentiated NCCIT cell lines expressing shRNA targeting GDF3 or a scrambled one (sh1GDF3 or scr). Both cell lines were successfully differentiated into neuronal progenitors. After 14 days of differentiation with retinoic acid (RA) the cells markedly changed their morphology from small, cobblestone-like cells with big nuclei, forming multilayer colonies to heterogeneous population of large, flat cells with long, branched neurites (Figure S3). TUBB3 immunofluorescence staining confirmed the neuronal differentiation, whereas the efficiency of the process in both cell lines was very similar (Figure 7A). The expression of paired box 6 (PAX6), nestin (NES), T-box 3 (TBX3) and TUBB3 as markers of neuronal differentiation, and OCT4, GDF3 and TDGF1 as markers of pluripotency was tracked in the course of differentiation by qPCR (Figure 7B). The time kinetic revealed that after 14 days the expression of investigated neuronal markers was similar. However, in the early phase of differentiation, especially on the day 4, significant differences could be observed. *NES*, *TBX3* and *TUBB3* were higher expressed in the GDF3 knockdown cell line at day 4. The expression of *PAX6* was already detected on day 4 in the sh1GDF3 cell line, but not in the control cells. Since NCCIT cells, as reported for many cancer cell lines [35]–[37], express basal level of *TUBB3*, the relative increase of *TUBB3* transcript number due to RA-treatment was only little, but significant. In the course of differentiation the pluripotency markers were downregulated. *OCT4* expression decreased similarly, independently of GDF3 expression status, while *TDGF1* downregulation occurred faster in sh1GDF3 cells. GDF3 was strongly downregulated in the control cells during first 4 days of differentiation.

Taken together, the expression pattern of differentiation and pluripotency markers suggest, that RA-induced differentiation occurs faster in the absence of GDF3.

### GDF3 knockdown enhances RA-induced apoptosis

**Figure 8:**
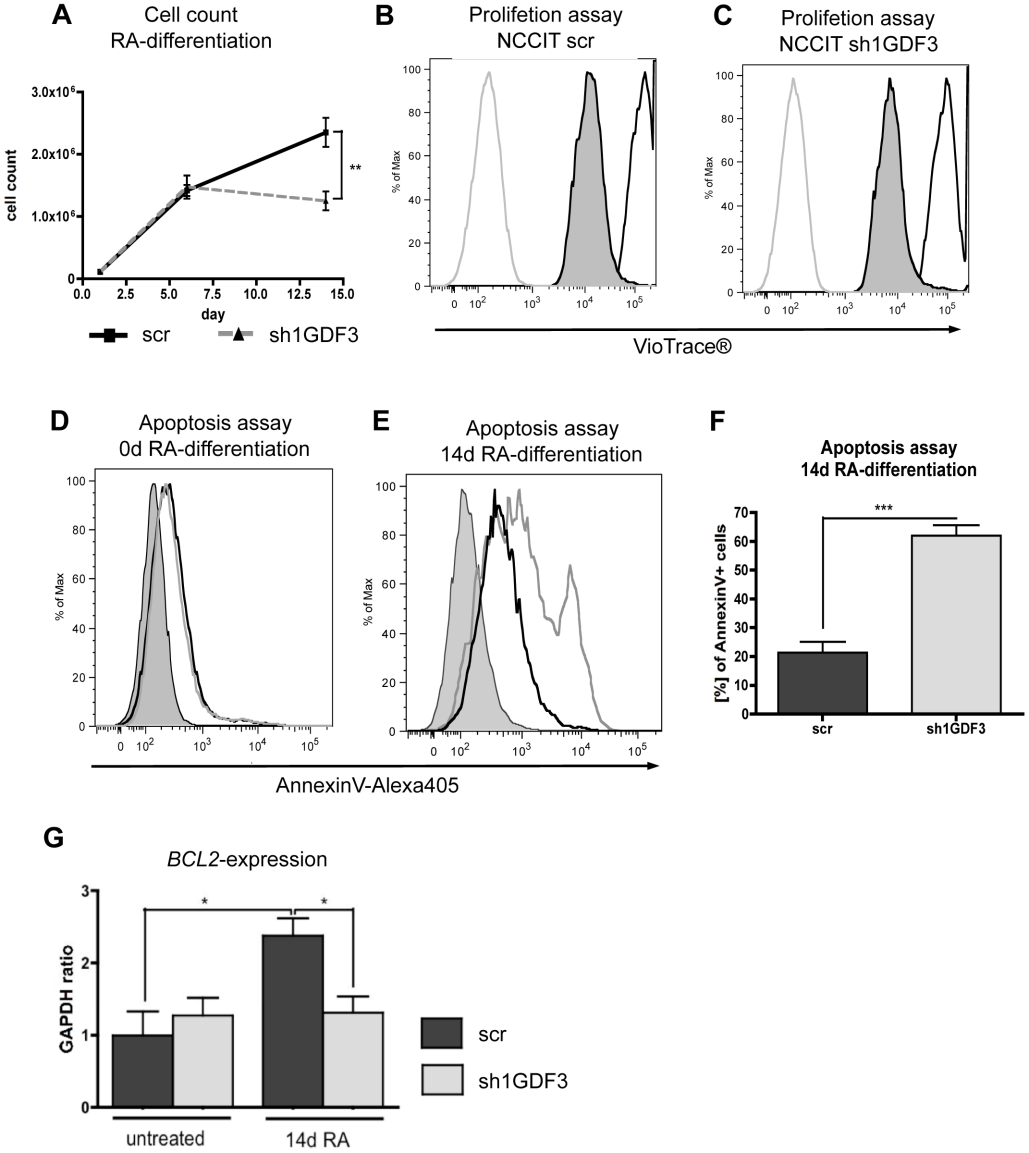
GDF3 knockdown mediates RA-induced apoptosis. **A.** Growth curve of NCCIT scr and NCCIT sh1GDF3 during RA-differentiation. n = 6 **B–C.** FACS analysis of the amount of CellTrace® Violet incorporated into 14-days RA-differentiated NCCIT scr (B) and NCCIT sh1GDF3 (C) cells directly after the staining (black line) and 48 h later (black line, shaded). The grey line represents unstained control. n = 3 **D–E.** AnnexinV-Alexa405 staining of NCCIT scr and NCCIT sh1GDF3 at day 0 (D) and day 14 (E) of RA-differentiation. The shaded curve represents unstained control. **F.** Quantification of E. n = 6 **G.** BCL2 expression in NCCIT scr and NCCIT sh1GDF3 before and after 14 d of RA-differentiation. The expression was measured by qPCR, the results are presented as GAPDH ratio and normalized to cells transduced with scrambled-vector. P-values smaller than or equal to 0.05 were considered significant. (*) indicates p≤0.05, (**) p≤0.01 and (***) p≤0.001. Data is represented as means +/− standard deviation. n = 3.

RA can trigger not only differentiation, but also apoptosis and growth arrest [38], [39]. During the RA-induced differentiation of NCCIT cells we observed significant difference in cell numbers between the GDF3-expressing and –knockdown cells. The growth kinetic in Figure 8A shows that after 14 days of differentiation the growth of control cell line slowed down, while cell number of sh1GDF3 cells did not increase from day 7 on. We investigated the proliferation after 14 days of RA-differentiation and observed the same rate in both cell lines (Figure 8B–C). As next step, we determined the apoptosis rate by Annexin V-staining. Both cell lines displayed the same rate of apoptosis before the RA-differentiation was initiated, as shown in Figure 8D. However, after 14 days of RA-treatment the number of apoptotic cells among GDF3-knockdown cells increased to 70% (Figure 8F) and was more than 2-fold higher than in the control cells. We extended the differentiation period for additional 14 days and observed that, on the contrary to NCCIT scr cell line, all the sh1GDF3 cells died (data not shown).

RA-induced apoptosis during neuronal differentiation can be prevented by transcriptional regulation of the expression of BCL2 family members [40], [41]. Therefore, we determined the expression level of several proteins from BCL2 family. Among the factors tested, only *BCL2* was 2-fold upregulated due to RA-treatment in NCCIT scr, but not in NCCIT sh1GDF3 cells. The expression of this pro-survival molecule remained unchanged during the course of differentiation in NCCIT sh1GDF3 cells and comparable to the level in untreated NCCIT scr cells (Figure 8G).

This data indicates that disruption of GDF3 pathway modulates the response of NCCIT cells to the differentiation cues *in vitro*, inducing apoptosis due to failure in BCL2 upregulation.

## Discussion

GDF3, as a pluripotency-associated factor and still an enigmatical member of TGFB family, has recently emerged as a new, potential player in cancer biology. Several research groups have already tried to decipher the role of GDF3 in ESC biology, breast carcinoma and myeloma, and have yielded contradictory results.

In this work, we present the first study to our knowledge investigating the role of GDF3 in cancer cells with stem-like characteristic. For this purpose we chose an embryonal carcinoma cell line, NCCIT cells. Embryonal carcinoma cells have already been recognized as a suitable cellular system for the study of CSC features because of an ES-like signature, high tumorigenicity and *in vivo* and *in vitro* differentiation capacity [1], [9], [10]. We confirmed that GDF3 and its receptors are expressed not only in NCCIT cells, but also in another, well-characterized embryonal carcinoma cell line NTERA2 (Figure S1). These two cell lines are the only pluripotent, human embryonal carcinoma cells lines isolated and reported in the literature so far. In most of the studies performed to date mouse cell lines (e.g. P19) were utilized. However, since GDF3 has been described to play a different role in mouse and human ES cells [13], it was essential to use human material in this work. Nevertheless, it will be necessary to validate the findings reported here in primary CSC. The fact that CSC can be detected and isolated, but no reliable protocol for their cultivation has been established so far makes this task very challenging.

In our study, we performed a comprehensive analysis of transcriptional effects of GDF3 in NCCIT cells. For this purpose we conducted a series of microarray experiments, comparing the effects of (1) elevated concentrations of GDF3 and (2) disruption of endogenous GDF3 signaling by lentiviral knockdown.

We found that GDF3 is strongly involved in cell signaling and developmental processes (Table 4). Especially in the latter group, genes related to the lineage commitment - ectoderm development and neurogenesis - were significantly overrepresented, when GDF3 signaling was disrupted or strongly enhanced by stimulation with a high GDF3 dose. Additionally, the GDF3 knockdown also affected genes related to mesoderm development. Our results led us to the conclusion that any perturbation of endogenous GDF3 signaling in our model of CSC drives the cells towards an exit from undifferentiated status and divergent differentiation programs. A similar course of events has been previously described for other members of TGFB family acting in ESC, such as ACTIVIN and NODAL [31], [41]. Furthermore, our results are in corroboration with those of Chen *et al.* [20] and Levine *et al.* [13], who obtained their data in ESC and showed that GDF3 is indispensable for normal development *in vivo* and *in vitro*.

The members of TGFB family are known to display differential effects depending on the concentration of the ligand [42]–[44]. Therefore, we tested transcriptional effects of different doses of GDF3 in NCCIT cells. From the data obtained in this study, we conclude that there is a common set of GDF3-specific target genes (Figure 3–5 and Table 5), from which several could be validated by qPCR. However, subtle changes in SMAD2/3 signaling strength can be sensed by the recipient cell and leads to alterations in the transcriptome, which might even result in the modulation of the direction of differentiation (Table 4). While a high concentration of GDF3 leading to strong activation of the signaling pathway(s) affects expression of genes related to development and lineage commitment, low concentration of the ligand predominantly impacts signal transduction processes (Figure 4A). In line with previous findings about other TGFB family members, this data hints at the capacity of GDF3 to act in dose-dependent manner [31]. Our findings about differential transcriptional responses to varying doses of GDF3 need to be complemented in future studies by the exact identification of target genes activated by low and high dose of GDF3.

We confirmed the role of GDF3 in developmental processes by validation of GDF3 targets emerging from the performed microarray experiments (Table 5). We considered homeobox genes as appealing targets, since they regulate not only embryonic development and tissue patterning, but also because their expression is frequently perturbed in tumors [44]–[46]. Therefore, we focused primarily on *HOXA9*, *HOXA10* and *HOXB13*. HOXA9 directly regulates BRCA1 expression, restricts breast tumor aggression and therefore elevated HOXA9 expression level can be correlated with optimistic prognosis for the patient [47]. GDF3 has been already reported to be expressed in breast cancer [15], although the expression level seem to be reduced in comparison to the surrounding, healthy tissue [14]. It would be interesting to investigate, whether the restoration of GDF3 level in breast cancer would increase HOXA9 expression and thereby reduce malignancy of disease and improve the clinical outcome for the patient. Interestingly, the expression of *HOXA9* in unstimulated, wildtype NCCIT cells was barely detectable, which led us to conclude, that endogenous GDF3 expression may not be high enough to robustly induce *HOXA9* expression and that exogenous introduction of the ligand is necessary.

Other highly interesting GDF3 targets were also reported to act as potent tumor suppressors. HOXA10 in breast cancer regulates p53 expression, modulate proliferation and invasiveness of breast cancer cells [48] and mediates differentiation in myeloid leukemic cells [49]. The expression of HOXB13 is known to be downregulated by hypermethylation of the promoter region in not only breast, but also colorecta [50], renal [51], prostate [52] cancer and melanoma [53]. Its reexpression diminishes tumor formation *in vivo* and *in vitro*.

Furthermore, the GDF3 stimulation experiment as well as GDF3 knockdown revealed involvement of GDF3 in the BMP-pathway. GDF3 treatment upregulated *BMPR2* (Figure 5C), while GDF3 knockdown diminished expression of several members of BMP-family, such as *BMP2*, *4* and *7* (Figure 5H). This GDF3-BMP link might be considered in designing anti-cancer treatment, since active BMP signaling in tumor has been reported to be beneficial for the disease outcome [54].

Due to technical limitations, GDF3 stimulation *vs.* GDF3 knockdown cannot be considered as complete counterparts in our experimental design. The stimulation was performed for 3 h, aiming to investigate short time, primary effects, avoiding effects of feedback loops and therefore delivering an insight into dynamically changing system. The GDF3 knockdown was performed via lentiviral delivery of a shRNA cassette, so that a stable GDF3 knockdown and control cell line were generated by expansion of transduced cells over several passages. Thus, it is not surprising, that the transcripts regulated by GDF3 stimulation and GDF3 knockdown have a common intersection but are not congruent.

The impact of GDF3 on the proliferation of NCCIT cells was also investigated in the scope of this study. However, contrary to the report published by Li *et al.* [18], GDF3 did not influence proliferation in our system. This discrepancy can possibly be attributed to the usage of different cell types.

Having confirmed the developmental role of GDF3 in our model of CSC, we treated NCCIT cells with retinoic acid *in vitro* to analyze, whether GDF3 knockdown would change the differentiation pattern. Despite a number of genes associated with neurogenesis regulated on the microarray (Table 4, Figure 3–4), we did not see any phenotypic differences between GDF3-knockdown cells and the control (Figure 7A & S3). However, the molecular analysis by qPCR revealed altered expression pattern during the RA treatment. The expression level of several differentiation and pluripotency markers in the early stage of the process hinted at accelerated neurogenesis in the absence of GDF3 (Figure 5B). This data is in accord with the study of Levine *et al.* [13], which demonstrated that overexpression of GDF3 can at least partially maintain the expression of pluripotency markers in hESC challenged with BMP4. Apparent contradiction to the report about the neuro-inductive potential of GDF3 in neuronal progenitor cells [18] may be due to usage of a different cellular system. A possible explanation is that the effect of GDF3 on cells already primed for differentiation can be strikingly different compared to uncommitted stem-like cells – a common phenomenon for the members of the TGFB family.

Retinoic acid is the only clinically available cyto-differentiating agent, employed in several cancer therapies, especially in treatment of acute promyelotic leukemia, but also of solid cancers, such as squamous cell carcinoma [55], neuroblastoma [56] and hepatocellular carcinoma [57] in humans and breast carcinoma [58] in mice. The concept for getting the malignant potential under control is forcing the tumor cells into finally differentiated status. However, the success of RA application in solid tumor was limited, because RA induces a variety of cellular programs depending on cell type, ranging from simple growth inhibition and differentiation to apoptosis.

Here, we report for the first time the influence of GDF3 on the outcome of RA-treatment of NCCIT cells *in vitro*. We made the unexpected observation, that under prolonged exposure to RA GDF3-knockdown NCCIT cells underwent apoptosis to much greater extent than their GDF3-expressing counterparts (Figure 8D–F). This data lead us to speculate, that GDF3 protects the CSC cells from RA-induced apoptosis. We were able to uncover at least one reason for this course of events. Our results indicate that the pro-survival effect of GDF3 is BCL2-dependent. *BCL2* was upregulated due to RA-treatment in control NCCIT cells, but not in GDF3-knockdown cells. However, it seems likely, that this is an indirect, secondary effect, possibly due to changes in the epigenetic landscape of GDF3-knockdown cells, since *GDF3* and *TDGF1* expression in the control cells was already downregulated in the early phase of RA-differentiation (Figure 7B). For future studies it might be of relevance to investigate the impact of GDF3 overexpression on RA-induced differentiation.

## Conclusions

In our study, we have confirmed GDF3 as an important player in cancer biology. Using NCCIT cells as a system with CSC-like properties we could demonstrate that GDF3 robustly induces expression of a panel of genes related to differentiation, among which are several potent tumor suppressors, such as *HOXA9*, *HOXA10* and *HOXB13*. This finding is important for development of new therapies against breast cancer.

Moreover, we report for the first time the protective effect of GDF3 against RA-induced apoptosis in CSC. Blocking of GDF3 combined with RA-treatment of solid cancers is a compelling direction for further investigations, which can lead to re-design of cancer differentiation therapies.

## Supporting Information

Figure S1 **Major components of GDF3 signaling pathway are expressed in NTERA2 cell line.**
(TIF)

Figure S2 **Knockdown of GDF3 by shRNA.** A. FACS analysis of the efficiency of lentiviral transduction detected by GFP expression. B. *GDF3* expression upon GDF3 knockdown with two different constructs sh1GDF3 and sh2GDF3. The expression was determined by qPCR, the results are presented as *GAPDH* ratio and normalized to scramled. n = 6.
(TIF)

Figure S3 **GDF3 knockdown does not change the morphology of RA-differentiated NCCIT cells.** Phase contrast (upper panel) and immunofluorescence photographs demonstrating morphological changes of NCCIT scr and NCCIT sh1GDF3 due to 14 d of RA-differentiation. GFP (lower panel) labels cells transduced with lentivirus delivering the shRNA-cassette. Black and white arrows indicate the characteristic features of neuron-like structures. Yellow bar indicates 100 µm. One representative example is depicted. n = 3.
(TIF)

Table S1 **Lists of biological processes to which genes regulated by GDF3 and subjected to the overrepresentation analysis were assigned.**
(XLSX)
