# Supplementary figures and images for: Growth and Differentiation Factor 3 Induces Expression of Genes Related to Differentiation in a Model of Cancer Stem Cells and Protects Them from Retinoic Acid-Induced Apoptosis
Source: PLoS One. 2013 Aug 12;8(8):e70612. doi: 10.1371/journal.pone.0070612 (PMC3741270; doi:10.1371/journal.pone.0070612)

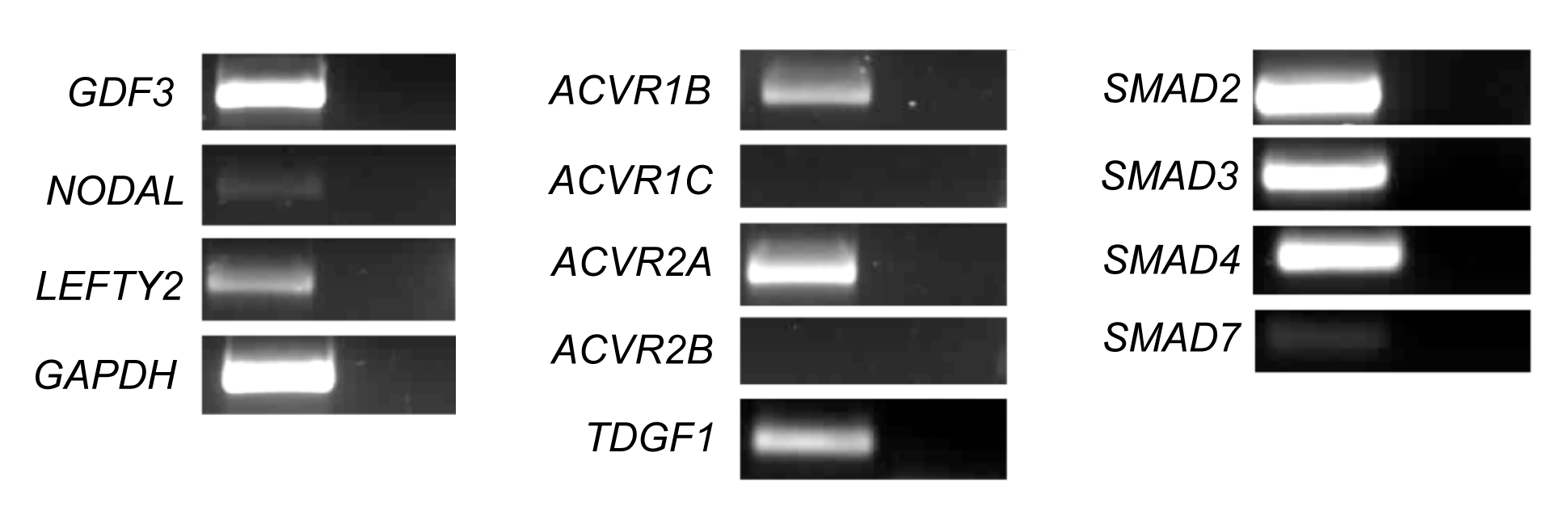

Supplement: Figure S1 — Major components of GDF3 signaling pathway are expressed in NTERA2 cell line. (TIF) [file pone.0070612.s001.tif]

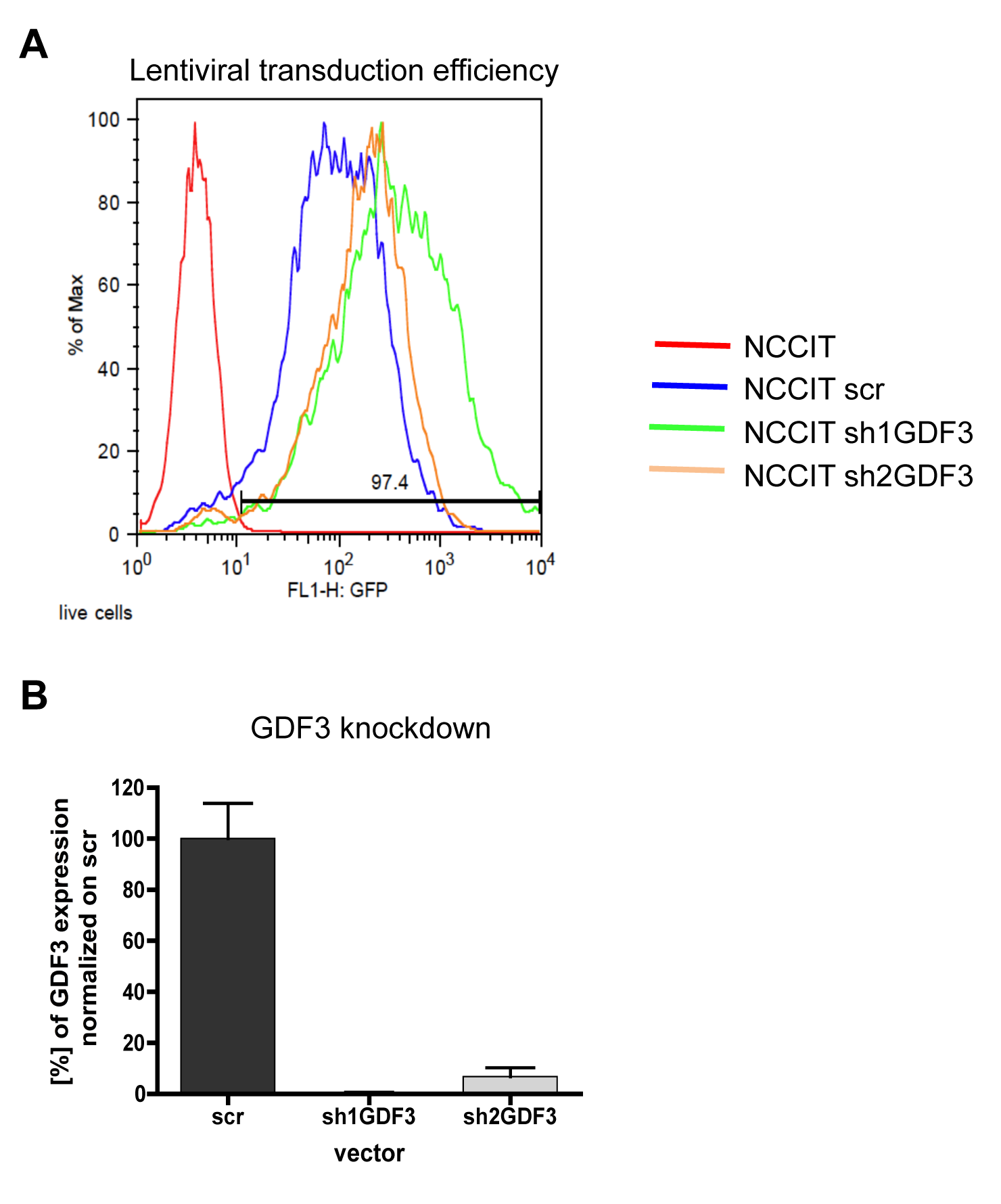

Supplement: Figure S2 — Knockdown of GDF3 by shRNA. A. FACS analysis of the efficiency of lentiviral transduction detected by GFP expression. B. GDF3 expression upon GDF3 knockdown with two different constructs sh1GDF3 and sh2GDF3. The expression was determined by qPCR, the results are presented as GAPDH ratio and normalized to scramled. n = 6. (TIF) [file pone.0070612.s002.tif]

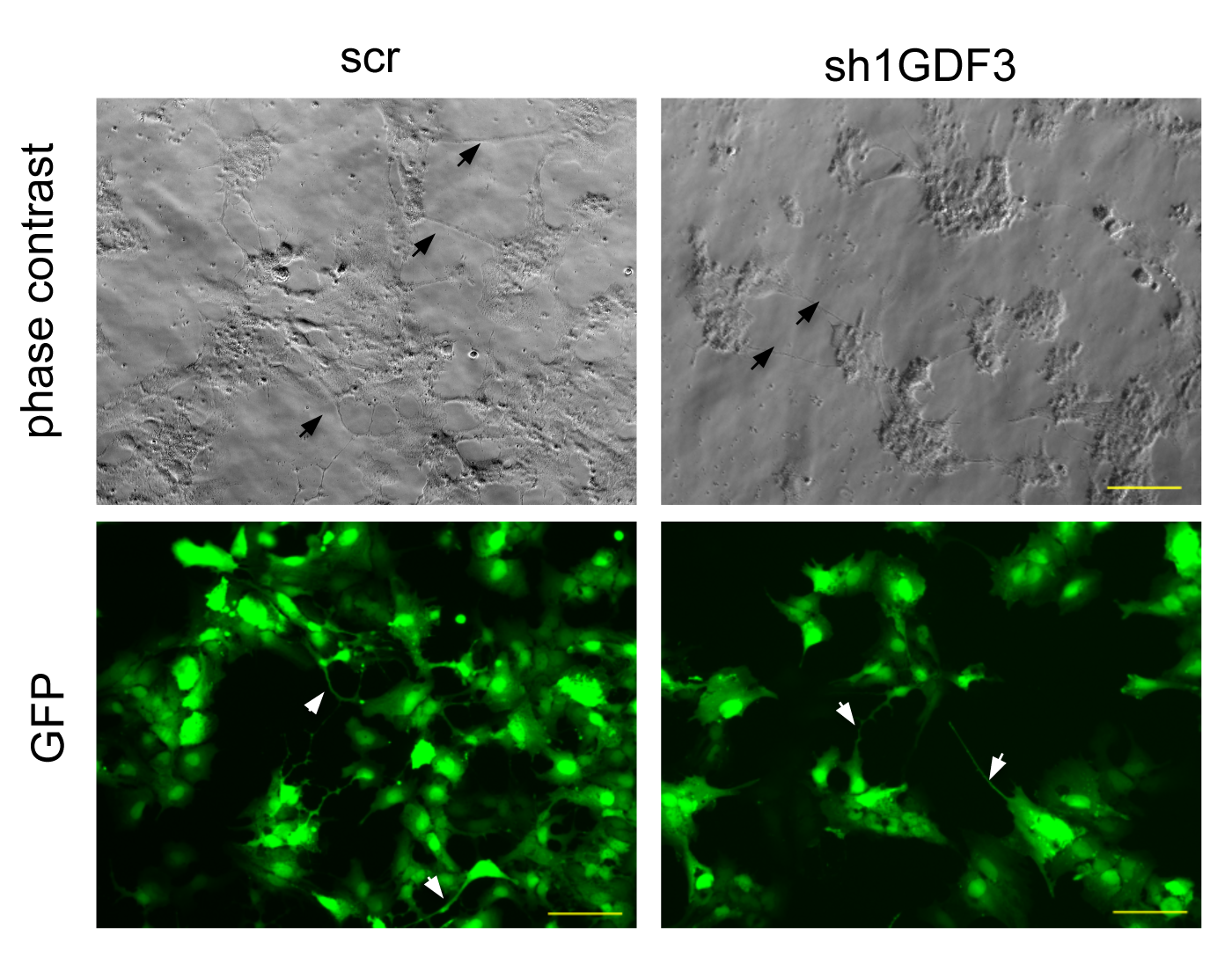

Supplement: Figure S3 — GDF3 knockdown does not change the morphology of RA-differentiated NCCIT cells. Phase contrast (upper panel) and immunofluorescence photographs demonstrating morphological changes of NCCIT scr and NCCIT sh1GDF3 due to 14 d of RA-differentiation. GFP (lower panel) labels cells transduced with lentivirus delivering the shRNA-cassette. Black and white arrows indicate the characteristic features of neuron-like structures. Yellow bar indicates 100 µm. One representative example is depicted. n = 3. (TIF) [file pone.0070612.s003.tif]
